# Ultrasensitive Dual ELONA/SERS–RPA Multiplex Diagnosis of Antimicrobial Resistance

**DOI:** 10.1021/acs.analchem.4c02165

**Published:** 2024-07-08

**Authors:** Waleed A. Hassanain, Christopher L. Johnson, Karen Faulds, Neil Keegan, Duncan Graham

**Affiliations:** † Department of Pure and Applied Chemistry, Technology and Innovation Centre, University of Strathclyde, Glasgow G1 1RD, U.K.; ‡ Translational and Clinical Research Institute, Newcastle University, Newcastle-Upon-Tyne NE2 4HH, U.K.

## Abstract

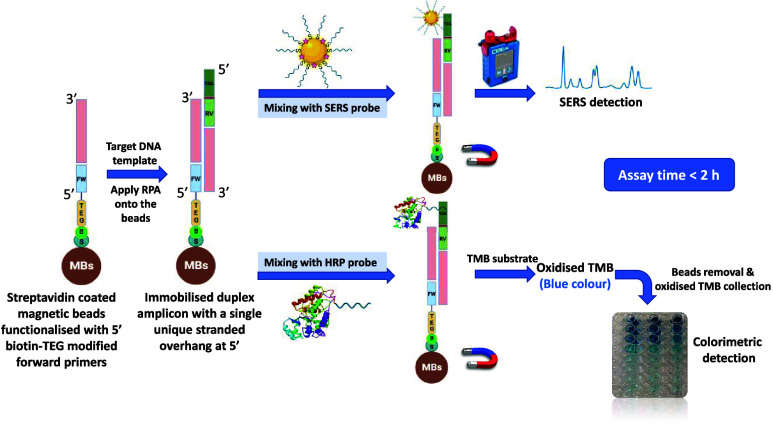

Antimicrobial resistance (AMR) is a significant global health threat concern, necessitating healthcare practitioners to accurately prescribe the most effective antimicrobial agents with correct doses to combat resistant infections. This is necessary to improve the therapeutic outcomes for patients and prevent further increase in AMR. Consequently, there is an urgent need to implement rapid and sensitive clinical diagnostic methods to identify resistant pathogenic strains and monitor the efficacy of antimicrobials. In this study, we report a novel proof-of-concept magnetic scaffold-recombinase polymerase amplification (RPA) technique, coupled with an enzyme-linked oligonucleotide assay (ELONA) and surface-enhanced Raman scattering (SERS) detection, aimed at selectively amplifying and detecting the DNA signature of three resistant carbapenemase genes, VIM, KPC, and IMP. To achieve this, streptavidin-coated magnetic beads were functionalized with biotin-modified forward primers. RPA was conducted on the surface of the beads, resulting in an immobilized duplex amplicon featuring a single overhang tail specific to each gene. These tails were subsequently hybridized with recognition HRP probes conjugated to a complementary single-stranded oligonucleotide and detected colorimetrically. Additionally, they underwent hybridization with similar selective SERS probes and were measured using a handheld Raman spectrometer. The resulting quantification limits were at subpicomolar level for both assays, allowing the potential for early diagnosis. Moreover, we demonstrated the platform capability to conduct a multiplex RPA–SERS detection of the three genes in a single tube. Compared to similar approaches like PCR, RPA offers advantages of speed, affordability, and isothermal operation at 37 °C, eliminating the need for a thermal cycler. The whole assay was completed within <2 h. Therefore, this novel magnetic scaffold ELONA/SERS–RPA platform, for DNA detection, demonstrated excellent capability for the rapid monitoring of AMR in point-of-care applications, in terms of sensitivity, portability, and speed of analysis.

## Introduction

Antimicrobial resistance (AMR) is considered a major global health and sustainable development threat. The World Health Organization (WHO) has identified AMR as one of the top 10 global public health threats facing humanity, and its impact is expected to worsen unless action is taken.^1^ The main cause of AMR can be attributed to the imprudent overuse of antimicrobials in humans and animals as well as in the surrounding environment. This, along with inadequate infection control policies, leads to the persistence of drug residues or resistant genes in the environment. As a result, microbes could adapt and find new ways to survive the effects of antimicrobials designed to kill them and become resistant, making the antimicrobials less effective.^2^ This has resulted in the emergence of new generations of “superbugs” that cannot be treated with existing medicines, potentially increasing the severity of the infection and affecting life expectancy.^3^ According to the U.K. Government Review on AMR,^3,4^ AMR-related infections are estimated to cause 700,000 deaths each year globally. This number is projected to rise to 10 million by 2050, along with a cumulative economic cost of $100 trillion.^3,4^ Failure to address the AMR problem has far-reaching consequences, resulting in significant costs, not only in terms of healthcare and finances but also with regard to the sustainability of our food supply, environmental health, and socioeconomic progress. To break the AMR cycle and protect the population health, no single action will provide an adequate solution. Urgent collaborative and coordinated efforts are required, for example, implementing infection prevention and control programs, as well as antimicrobial stewardship initiatives in healthcare settings, strengthening the tracking systems, and raising awareness about the reasonable use of antimicrobials. Additionally and importantly, enhancing the capability of laboratories to develop in-field analysis and point-of-care (POC) diagnostic tests that can report resistant pathogenic strains in a reasonable time, especially in limited resource settings, will play an important role.^5^

Apart from traditional time-consuming culture methods,^6^ molecular methods including DNA microarrays, metagenomics, and the polymerase chain reaction (PCR) have been used extensively as detection techniques for AMR diagnosis.^7^ While DNA microarray and metagenomics are valuable tools for research and understanding of the microbial communities in various environments, they are not practical for in-field and POC clinical diagnostics. They involve extensive sample processing, DNA extraction, sequencing, specialized equipment, and bioinformatics analysis, which are typically carried out in a well-equipped laboratory setting.^7^ PCR is the gold-standard nucleic acid technique for identifying resistant genes.^8^ It has the ability to provide rapid and precise amplification of specific DNA sequences, making it a valuable tool in various applications, including clinical diagnostics and genetic research.^9^ However, it requires temperature cycling in a well-controlled environment, which limits its application for in-field and POC testing.^8^ To overcome this limitation, isothermal amplification techniques, like recombinase polymerase amplification (RPA), have been developed and used as a successful alternative tool for PCR in several diagnostic applications.^8,10,11^ Compared to PCR, RPA is a faster, cheaper, and isothermal technique that operates optimally at 37 °C, which eliminates the need for a thermal cycler.^12^ In addition, RPA is a highly sensitive technique. Furthermore, POC-compatible readouts, such as portable spectrometers and equipment-free naked-eye strategies, could be integrated with RPA to enable its rapid in-field diagnostic applications. These characteristics position RPA as a promising option for POC applications.^8^ However, using RPA alone might not be the optimal approach to conduct multiplex assays, which are often essential to provide more comprehensive information about resistant biomarkers. Therefore, integrating RPA with another rapid and highly sensitive diagnostic method, which supports single-tube multiplex RPA detection, has the potential to improve the overall performance of RPA in the broader field of POC diagnostics.^8,10,11^ Surface-enhanced Raman scattering (SERS) is a powerful analytical molecular spectroscopy technique.^13−16^ It has generated precedential attention as a highly promising readout technology for rapid diagnostic assays. Besides its inherent sensitivity, the narrow and distinct spectral peaks of SERS make it ideal for applications demanding multiplexing analysis.^17,18^ The utilization of nanostructures labeled with different Raman reporters has enabled a range of multiplex detection applications, especially in clinical diagnostics.^19,20^ Additionally, SERS is considered as a technique that strongly aligns with in-field and POC testing.^21,22^ Therefore, the synergistic combination of SERS with RPA in one platform provides a significant benefit in the development of a powerful multiplex diagnostic system for AMR at POC.^8,10,11^

In a recent application,^23^ we reported a solid-phase RPA assay conducted within spatially separated streptavidin-coated microplates for the detection of the big five carbapenemase genes, as demonstrators for the burden of bacterial AMR. While the application of resonance Raman spectroscopy as a detection mode improved the detection sensitivity over colorimetry readout, down to femtomolar level, the method was only able to detect each gene individually in a singleplex mode for parallel detection. The study concluded with a forward-looking scope, aiming to transfer the RPA assay from a solid-phase assay in a microplate to a solid-phase assay employing magnetic beads coupled with a SERS readout. This transition was intended to enable the highly sensitive detection of each gene in a true single-tube multiplex assay. Herein, we demonstrate a novel proof-of-concept in situ RPA protocol directly on the surface of a magnetic bead for the dual ELONA/SERS detection of the DNA signatures of the carbapenemase genes. The genes examined included verona integron-encoded metallo-β-lactamase (VIM), Klebsiella pneumoniae carbapenemase (KPC), and imipenemase-type metallo-β-lactamase (IMP). Additionally, the platform was utilized to conduct a multiplex RPA–SERS detection of the three genes in a single-tube test. To the best of our knowledge, this is the first report of DNA amplification using in situ RPA directly on the surface of a magnetic bead and not in a separate reaction. This not only enhances the speed of the assay but also minimizes sample loss during subsequent transfer and interaction processes with the magnetic beads. Additionally, the combination of RPA with a handheld Raman spectrometer offers the potential to decentralize AMR diagnosis, bringing it from centralized laboratories to POC and in-field analysis, thus facilitating the rapid acquisition of test results and enabling timely medical intervention to combat AMR. Furthermore, reducing sample backlogs and the associated biological risks linked to the transfer of resistant gene samples between different laboratories.^24^ Moreover, the use of the same platform to enable dual parallel independent readout modes, SERS and ELONA, is of significant importance in terms of cost-effectiveness of the assay, confirmation, and cross-validation of the results.^25^

## Experimental Section

### Preparation of Functionalized Magnetic Beads

A 500 μL aliquot of streptavidin-coated magnetic beads was placed in a LoBind tube. The beads were washed three times with the aid of a magnetic stand using 1 mL of PBST (1× PBS + 0.05%_(v/v)_ Tween-20). The washed beads were then functionalized with 5′ biotin-TEG-modified forward primer (VIM, KPC, or IMP) before the addition of 1 mL of PBST. The tube was then removed from the magnetic stand and placed in a rotating carousel for 1 h at room temperature to allow the primer to bind. The tube was then removed from the carousel and placed in a magnetic stand, and the beads were washed three times using 1 mL of PBST to remove the excess primer. The washed beads were then resuspended in 250 μL of distilled H_2_O. Accordingly, a 4 μL aliquot of the final prepared stock will contain 13.25 pM of 5′-functionalized biotin-TEG forward primer. Samples were then stored at 4 °C.

### RPA on Functionalized Magnetic Beads

RPA liquid basic kits were used for the assays, and the master mix was prepared as per the manufacturer’s instructions. Briefly, per reaction, 25 μL of 2× reaction buffer, 3.6 μL of dNTP mix (25 mM), 5 μL of 10× Basic E-mix, 4 μL of functionalized magnetic beads, 1.2 μL of reverse primer (10 μM), 1.2 μL of distilled H_2_O, 2.5 μL of 20× core reaction mix, and 2.5 μL of magnesium acetate (280 mM) were used. The RPA master mixes were prepared accordingly based on the above single reaction volumes. 45 μL of the prepared master mix was added to a 96-well plate mounted on top of a magnetic stand to secure the magnetic beads to the bottom of the well-plate, with wells containing either 5 μL of template for sample reactions (5 fg to 50 ng) or 5 μL of distilled H_2_O for non-template control (NTC) reactions. The 96-well plate and magnetic stand were then shaken for 1 min at 300 rpm using a plate shaker before being placed in a 37 °C oven for 40 min with occasional shaking. The plate and magnetic stand were then removed from the oven, and the samples were washed four times using 150 μL of PBST per well for 5 min while the plate was shaken at room temperature. Post-amplification reactions, the beads were resuspended in 100 μL of PBST for subsequent ELONA and SERS analyses.

### Preparation of the Selective SERS Probes

Selective SERS probes for each target were prepared by functionalizing Raman reporter-labeled gold nanoparticles with the corresponding ssDNA for each gene, as described in Table S1, Electronic Supporting Information (SI). The three prepared SERS probes were then characterized using extinction spectroscopy and dynamic light scattering (DLS) measurements (Figures S1 and S2, SI, respectively).

### Dual ELONA/SERS–RPA Measurements

To the amplified target templates on the magnetic beads surface, selective HRP/SERS probes (Table S1, SI) were added in the presence of a hybridization buffer and incubated for 30 min at room temperature while shaking.

### Control Study

To confirm the specificity of the HRP/SERS probes toward their targets, both probes of each target were tested against a high concentration of different genes.

### Multiplex SERS–RPA Measurements

In order to perform a multiplex SERS detection of the three genes, 8 different mixtures of the amplified target-carrying beads were prepared (samples 1–8, Table S2, SI).

A detailed experimental procedure is included in the SI.

## Results and Discussion

### RPA on the Surface of Magnetic Beads

**Figure 1:**
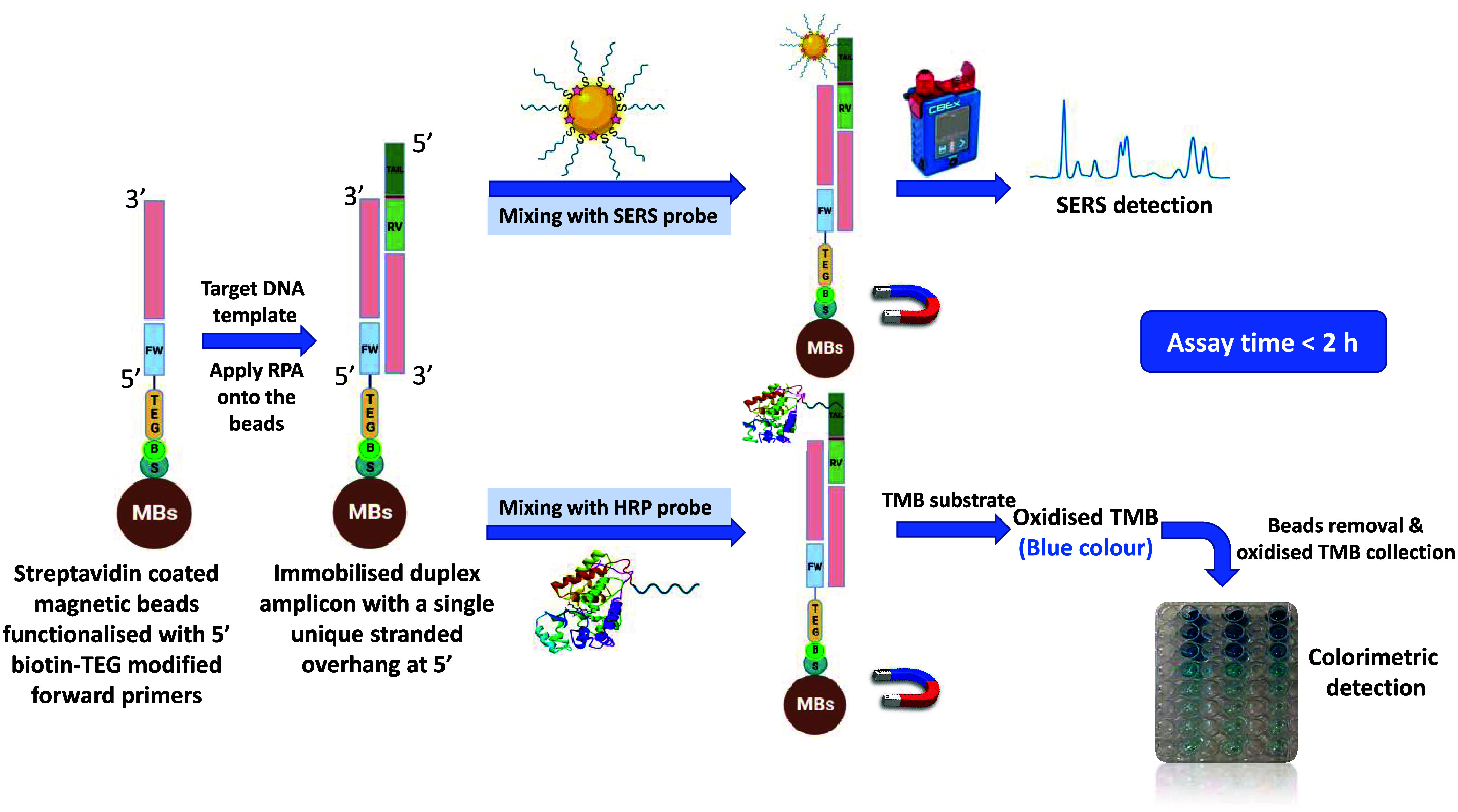
A schematic illustration for the dual ELONA/SERS–RPA sensing platform for AMR detection.

Magnetic beads-based amplification applications have become increasingly important in molecular diagnostics, especially for the detection of infectious diseases.^26^ These methods utilize magnetic beads coated with specialized probes or primers to detect specific nucleic acid sequences of interest. They provide several benefits in terms of the ability to detect multiple targets simultaneously, high levels of sensitivity and specificity, user-friendly automation, adaptability, and stability. The working principle of this new assay format is illustrated in Figure 1. It consisted of streptavidin-coated magnetic beads functionalized with a biotin-modified forward primer, with RPA performed on the surface of the beads. The resulting product was an immobilized duplex amplicon with a single unique overhang tail at the 5′ position of each gene, imparted by a tailed reverse primer. This tail was then hybridized with a horseradish peroxidase (HRP) probe tethered to a complementary DNA sequence and detected colorimetrically. In a parallel test, the gene-specific tail was also hybridized with a selective SERS probe and measured using a handheld Raman spectrometer for SERS detection. The oligonucleotides sequence of the HRP and SERS probes of each gene is described in Table S1 (SI). The sequence of the forward primers and the tailed reverse primers for each target gene is described in our previous study.^23^ The RPA procedure operates optimally at 37 °C, thus eliminating the need for a thermal cycler. Additionally, the in situ RPA directly on the surface of magnetic beads eliminated the need for a separate reaction. Thus, the assay process is accelerated while reducing sample loss during transfer and interaction with the beads.

### Synthesis of SERS Probes

In this work, selective SERS probes were developed and used as detection probes for resistant genes. These probes were used to improve the specificity and sensitivity performance of the assay in a quantitative manner. To ensure consistent enhancement of the SERS signal, a meticulous design was applied to the SERS probes. Each gene SERS probe was prepared in a similar manner, utilizing distinct complementary recognition ssDNA for each target. AuNPs were chosen as the enhancement source due to their notable stability against oxidation, high extinction cross section in the visible spectral range, and their facile functionalization with DNA through established reactions.^18,27−29^ For the AuNPs labeling, different Raman reporters were carefully evaluated to facilitate the subsequent multiplex SERS detection of the three genes without significant overlap from the various bands in the spectrum. For this purpose, 4-(1*H*-pyrazol-4-yl)pyridine (PPY), 1,2-bis(4-pyridyl)ethylene (BPE), and 4,4′-dipyridyl (DIPY) were chosen as Raman reporters due to their robust binding to AuNPs via the nitrogen atom in their pyridine ring structure, resulting in substantial signal enhancement and distinctive spectral features.^30^ Additionally, these reporters enabled the simultaneous detection of the genes by monitoring the unique diagnostic Raman peaks associated with each SERS probe, which correspond to each gene when mixed in a single tube.

Several strategies have been tested to attach the thiolated ssDNA onto the AuNPs, as well as to protect the colloidal stability of the AuNPs while maintaining the function of the attached DNA. For example, the salt aging protocol enabled the creation of durable probes. However, its protracted nature, involving a gradual NaCl addition over 2 days, proved to be impractical and could potentially affect the workflow efficiency.^31^ Accordingly, we shifted to use the fast acidic pH-assisted method, which involves the use of acidic sodium citrate buffer (pH 3) to facilitate the rapid adsorption of the DNA loading onto the AuNPs surface.^31,32^ At this low pH, the charge density of the AuNPs decreases, leading to protonation of some DNA bases such as adenine and cytosine. This reduces the charge repulsion between ssDNA and AuNPs, as well as among DNA strands themselves. However, the addition of NaCl salt remains necessary because both ssDNA and AuNPs retain some negative charges, even at a pH of 3.^33,34^ The prior manipulation of the solution with thiolated ssDNA at a pH of 3 facilitated the rapid administration of a high concentration of NaCl without causing any aggregation of the nanoparticles. Accordingly, this combined approach of utilizing low pH and salt solutions improved the functionality of the SERS probes, resulting in the adsorbed ssDNA being oriented in a standing conformation, which improved the overall hybridization capability of the probes while maintaining their stability.^31,35^ The conjugation of the thiolated ssDNA onto the AuNPs surface was monitored by comparing the extinction spectra and DLS measurements of the bare AuNPs and the SERS probes of the three genes. As shown in Figure S1 (**ESI**), band shifts ranging from 1 to 3 nm were observed in the AuNPs extinction spectrum after producing the SERS probes for the three genes. These shifts were attributed to the increase in the AuNPs size. The DLS measurements showed that the average size of the AuNPs increased from 63 nm to 88, 80, and 86 nm after the synthesis of the SERS probes of VIM, KPC, and IMP, respectively (Figure S2, SI). These experimental findings can be used as evidence for the successful attachment of the thiolated ssDNA and Raman reporters onto the AuNPs surface.

### Quantitative ELONA/SERS–RPA Detection of the Resistant Genes

**Figure 2:**
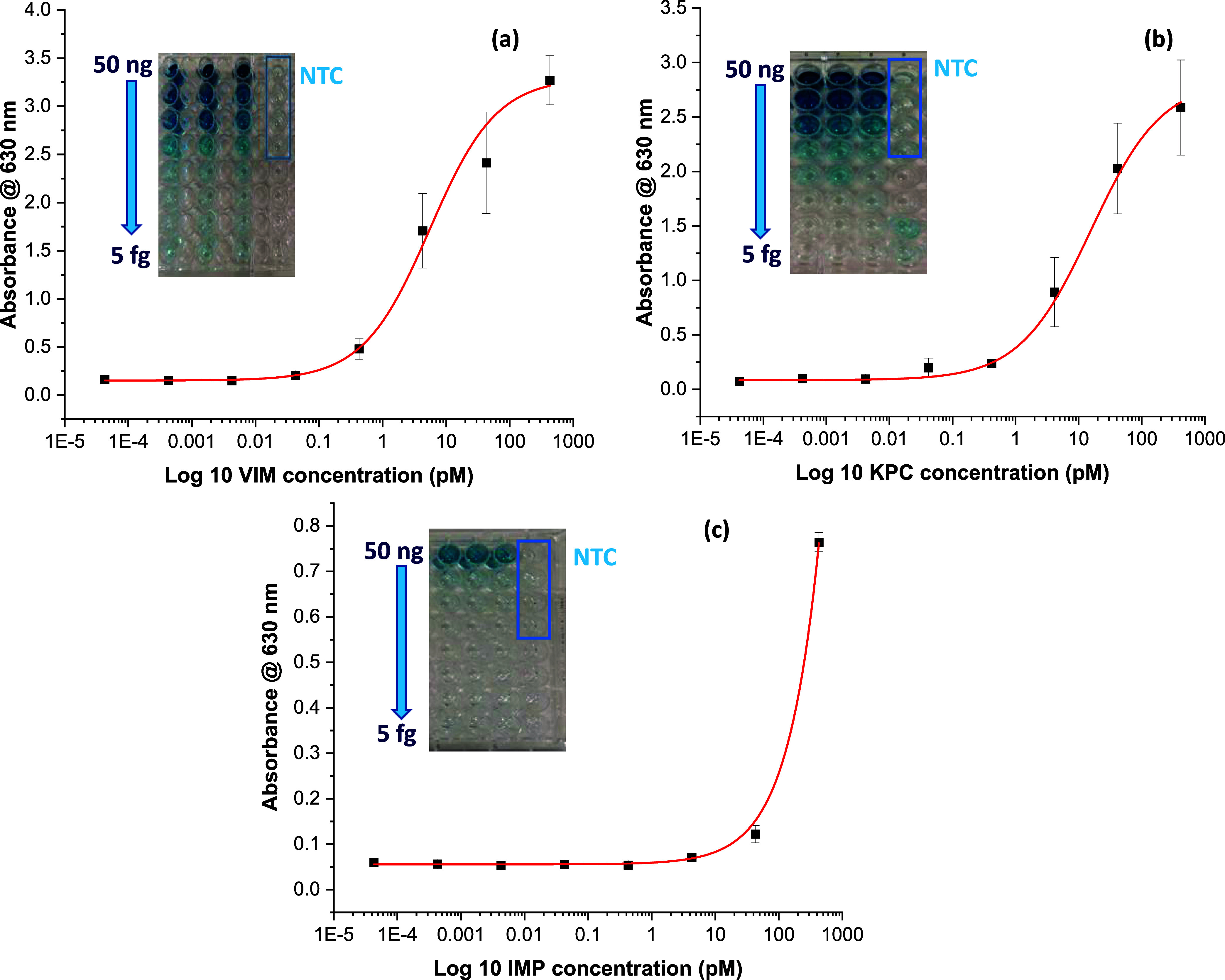
ELONA calibration curves for (a) VIM, (b) KPC, and (c) IMP. The insets show photographic images for the resulting blue-colored solutions after performing the RPA step. All curves were fitted to a logistic sigmoidal fitting algorithm. Error bars indicate the standard deviation from three measurements. All of the colorimetric measurements were carried out using a microplate reader, and the absorbance was measured at 630 nm.

The principle of this dual combined detection approach relies on the formation of a sandwich-like structure. This structure occurs via a DNA–DNA hybridization event between the tailed amplicon onto the magnetic beads surface and the cognate HRP/SERS probes. Subsequently, each sandwich-like structure was magnetically separated from the solution. For ELONA, the extracted beads were combined with the TMB substrate. Upon its oxidation, the substrate yielded a radical cation in equilibrium with a charge-transfer complex (CTC). This CTC molecule has an electronic transition with a λ_max_ of 650 nm and gives rise to a blue color change.^23,36^ The resulting blue solutions were then separated from the magnetic beads to prevent interference during colorimetric detection. It can be observed by eye that the intensity of the blue color increased with increasing template concentration in the RPA reaction. This effect was particularly noticeable with VIM and KPC and to a lesser extent with IMP, as shown in Figure 2. In the absence of the target template, no RPA takes place, preventing the formation of the 5′ overhang imparted by the reverse primer. Accordingly, no blue color solution should be formed. This is referred to as the non-template control (NTC) sample. The developed blue-colored solutions were scanned by a microplate reader, and their mean absorbance readings (*n* = 3) at 630 nm were plotted against the Log 10 value of various concentrations of the RPA-amplified targets (5 fg to 50 ng) to construct ELONA calibration curves (Figure 2a–c). As a nonlinear relationship was obtained between the different concentrations of the RPA-amplified templates and the absorbance values, a logistic sigmoidal fitting algorithm was employed in plotting the calibration curves.^23,37−39^ The *R*-squared values for all curves were >0.95, and the limits of quantification (LOQs) were 0.01, 0.45, and 7.59 pM for VIM, KPC, and IMP, respectively. The calculations of the conversion of the mass concentrations to the equivalent molar concentration values are described in the SI.

**Figure 3:**
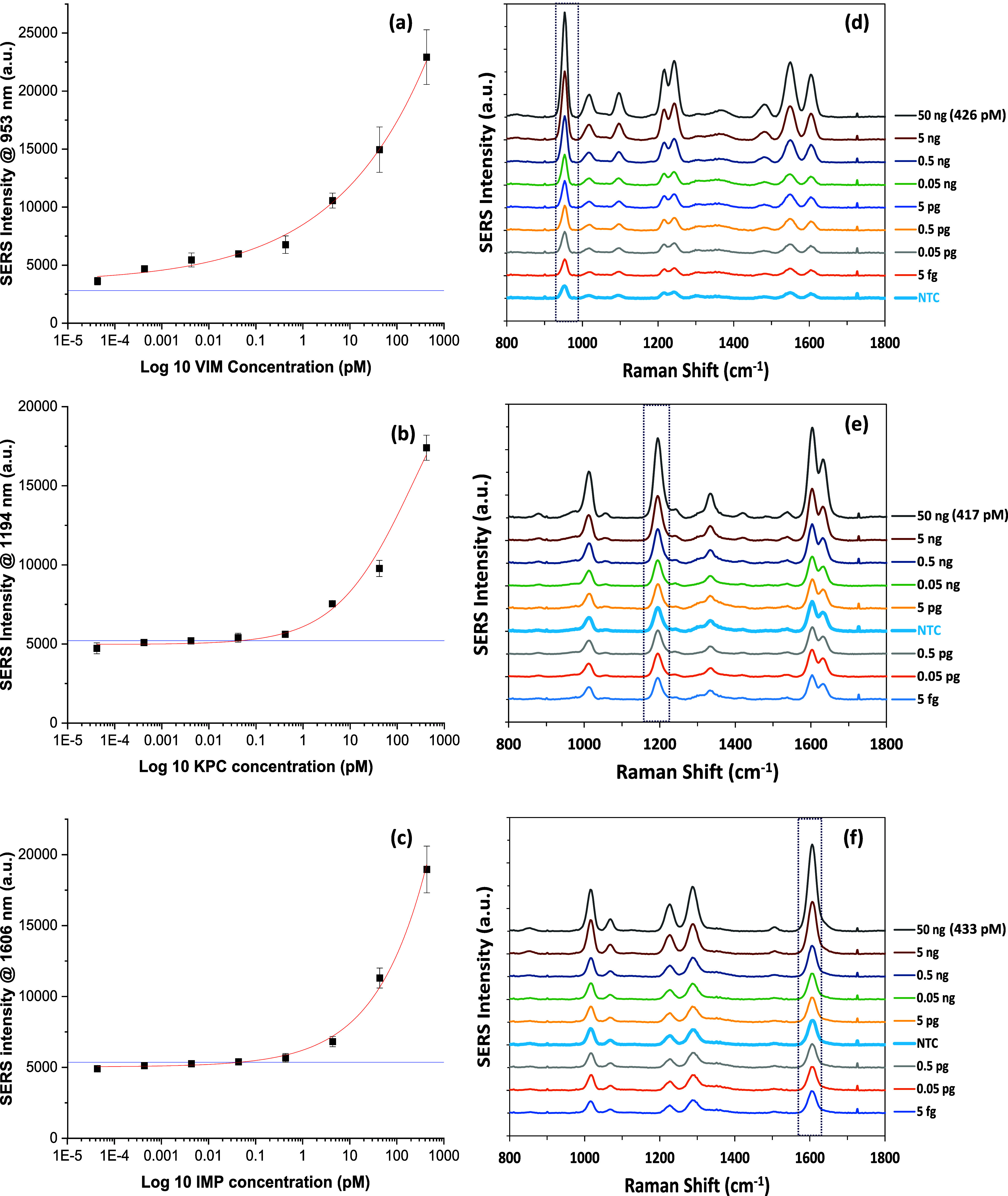
SERS calibration curves for (a) VIM, (b) KPC, and (c) IMP. The blue horizontal lines in each curve represent the NTC value that was used for LOQ calculation for each gene. LOQ was calculated using the formula: average NTC readings (*n* = 3) + (10× standard deviation of NTC readings). The corresponding SERS spectra for (d) VIM, (e) KPC, and (**f**) IMP in the range of 5 fg to 50 ng. All curves were fitted to a logistic sigmoidal fitting algorithm. Error bars indicate standard deviations from three measurements. All of the SERS measurements were carried out using a handheld Raman spectrometer equipped with a 785 nm laser excitation source at a 45 mW laser power with an acquisition time of 1 s at ORS mode.

For the SERS quantification of the RPA-amplified targets, the sandwich-like structure on the beads surface, containing a SERS probe specific to each target, was washed with 0.1 M PBS to remove the excess unbound probes from the beads surface. The interaction between the RPA-amplified targets on the beads surface and the SERS probes was monitored by scanning electron microscope images (Figure S3, SI). Subsequently, the beads were resuspended in 0.1 M PBS buffer and measured using a handheld Raman spectrometer for 1 s in an orbital raster scanning (ROS) mode to acquire the average SERS signal from each solution and reduce variability in the SERS signal across repeated scans.^40,41^ This facilitated the highly sensitive quantitative detection of each target. The mean SERS signal intensity (*n* = 3) of the peaks at 953, 1194, and 1606 cm^–1^, corresponding to the SERS probes of VIM, KPC, and IMP, respectively, was recorded and used to construct SERS calibration curves for each target (Figure 3a–c). It was observed that the mean SERS signal intensity increased gradually with increasing target concentrations in the range of 5 fg to 50 ng (Figure 3d–f). Similar to plotting the ELONA curves, a sigmoidal fitting algorithm was employed in plotting the SERS calibration curves.^23^ The *R*-squared values for all curves were >0.96, and the LOQs were 0.01, 0.26, and 0.12 pM for VIM, KPC, and IMP, respectively. In ELONA/SERS measurements, VIM demonstrated the lowest LOQ value among the other genes, which is reflective of low NTC absorbance and SERS signal values during the assay. This is attributed to a minimal primer dimer formation during the amplification.^23,42^ The NTC values were greater for both the KPC and IMP assays. This suggests that these primer sets have a greater propensity for primer dimerization, which brings about a concomitant increase in the NTC values, provided the reverse primer tail is still free to bind its reporter probe post-dimerization.^23,42^

These findings indicate that ELONA and SERS calibration curves generated for each gene are following a similar trend and validating each other. Additionally, this novel proof-of-concept dual combined ELONA/SERS–RPA approach exhibited significant potential for the ultrasensitive, early, and rapid (<2 h) diagnosis of AMR through the sensing of the DNA signature of resistant genes. Moreover, the use of the same platform to enable two independent readout modes conferred considerable benefits in terms of potential assay cost, result confirmation, and cross-validation. Furthermore, the utilization of a handheld Raman spectrometer to collect the results demonstrates the capability of the developed platform to be adopted for in-field testing of AMR in a simple and cost-effective way, as well as for the rapid acquisition of the test results.^43^

### Selectivity of HRP/SERS Probes and SERS Measurement Reproducibility

To evaluate the selectivity of our methodology, we conducted negative control tests for both the HRP and SERS probes against high concentrations of the cognate and non-cognate amplified target templates (Figure S4, SI). Additionally, to ensure the consistency of the SERS measurements, we conducted a series of 10 separate SERS measurements on different days, utilizing distinct batches of the synthesized SERS probes (Figure S5, SI).

### Multiplex SERS Detection

**Figure 4:**
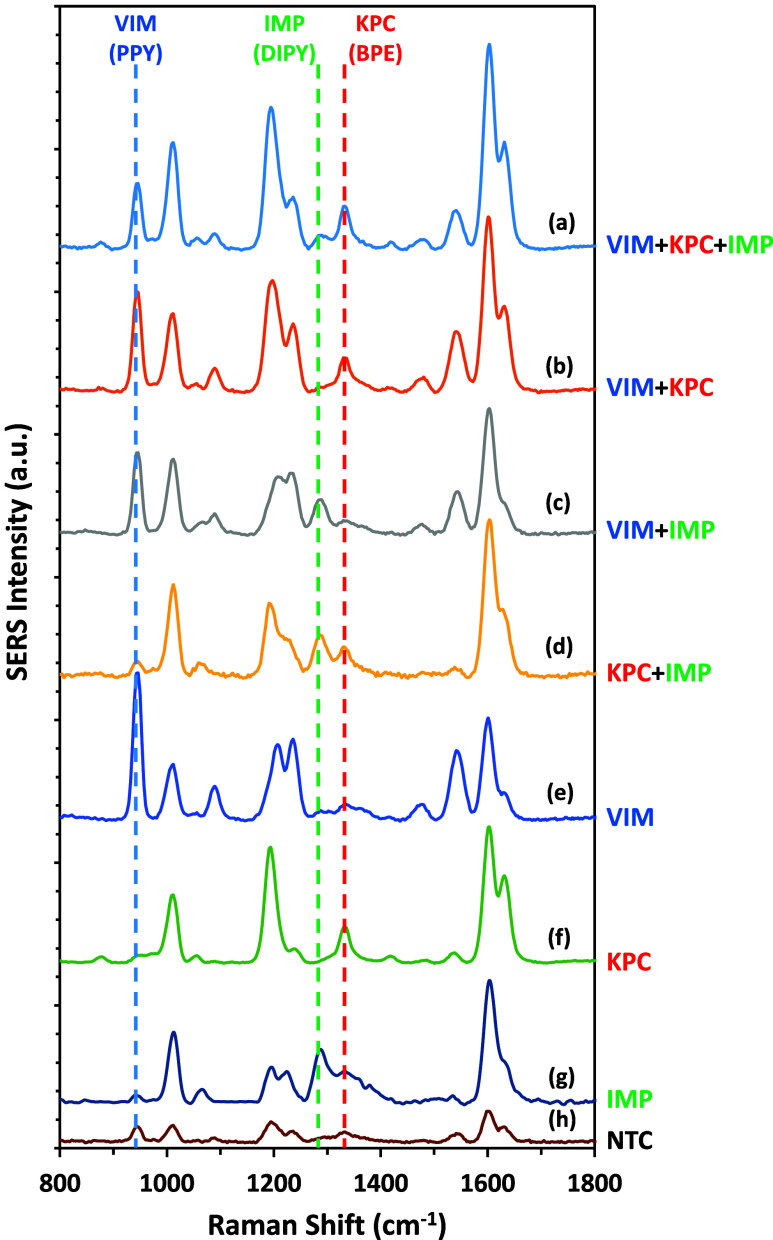
SERS spectra obtained from the multiplex testing for the eight probability samples (a) VIM + KPC + IMP, (b) VIM + KPC, (c) VIM + IMP, (d) KPC + IMP, (e) VIM, (f) KPC, (g) IMP, and (h) NTC. The blue, green, and red dotted lines show the characteristic diagnostic peaks for VIM, IMP, and KPC SERS probes, respectively, and hence are used to identify the corresponding gene. All of the SERS measurements were carried out using a handheld Raman spectrometer equipped with a 785 nm laser excitation source at a 45 mW laser power with an acquisition time of 1 s at ORS mode.

Multiplex detection of resistant genes in a “one-pot” test is critical in the ongoing battle against AMR. It improves the diagnostic process, saving precious time and resources, while guaranteeing comprehensive treatment strategies tailored to the specific resistant genes present.^44^ The sharp and unique molecular fingerprint spectroscopic peaks of SERS facilitate its exceptional simultaneous detection capability of multiple targets within a single sample.^45^ In this test, the SERS probes for each gene were combined together to create one multiplex SERS probe. Each probe exhibits a unique diagnostic peak corresponding to the Raman reporter that it contains. When combined together, VIM, KPC, and IMP SERS probes have their Raman reporters showing their characteristic peaks at 953, 1335, and 1291 cm^–1^, depicted as blue, green, and red dotted lines, respectively in Figure 4. The multiplex SERS probe was tested against eight samples simulating eight different infection scenarios with single, duplex, and triplex resistant genes, as well as NTC samples in a “one-pot” test. The concentrations of each gene per each sample are summarized in Table S2, SI. When the multiplex SERS probe was tested against a triplex target template mixture, the resulting spectrum displayed the three diagnostic peaks of the Raman reporters (Figure 4a). This triplex spectrum was compared to the SERS spectra obtained from the other seven samples (single, duplex, and NTC). After testing the multiplex SERS probe against duplex samples, only the corresponding two Raman reporters and their diagnostic peaks were evident in the resulting spectra, with minimal or even no SERS signal contribution from the third reporter of the multiplex SERS probe. This is attributed to the removal of the third individual SERS probe from the sample matrix after washing due to the high selectivity of each SERS probe as discussed previously. As indicated in Figure 4b–d, the SERS spectra represent (VIM + KPC), (VIM + IMP), and (KPC + IMP) duplex target template mixtures, respectively, after testing with the multiplex SERS probe. When the multiplex SERS probe was tested against single target template samples, only the SERS signal of the corresponding Raman reporter was reflected in the spectra due to the removal of the other two individual SERS probes from the sample matrix after washing. As illustrated in Figure 4e–g, the SERS spectra represent VIM, KPC, and IMP single target template samples, respectively, after being tested against the multiplex SERS probe. Testing the multiplex SERS probe with a triplex NTC template mixture yielded a poor SERS signal compared to the other seven samples (Figure 4h). Overall, the results obtained from this multiplex testing system demonstrate the proof-of-concept capability of our SERS–RPA testing platform to concurrently identify multiple resistant genes. The use of a handheld Raman spectrometer for collection of the results showcases the adaptability of the developed platform for on-the-go diagnostics in a user-friendly way, offering a more expedited and straightforward approach.^46^

## Conclusions

In this work, we introduced a proof-of-concept magnetic scaffold dual ELONA/SERS–RPA multiplex sensing platform for AMR diagnosis within <2 h. In comparison to routine PCR, the established gold-standard technique for AMR detection, RPA is faster, more affordable, and an isothermal amplification technique. The innovative nucleotide amplification strategy using in situ RPA on the magnetic beads surface improved the assay speed while minimizing any potential sample loss during subsequent transfer and interaction with the beads in a separate test. The use of a single platform to enable two independent readouts, SERS and ELONA, holds considerable significance for the assay in terms of cost-effectiveness and cross-validating the results. Furthermore, we showcase the platform’s capability to perform a multiplex RPA–SERS detection of eight different AMR infection scenarios in a “one-pot” test. The integration of RPA with a handheld Raman spectrometer, instead of using benchtop devices, holds promise for decentralizing AMR diagnosis, as well as enabling fast test result collection. The real-time analysis of samples coupled with the high sensitivity, selectivity, and reproducibility of the ELONA/SERS–RPA platform paves the way toward an accurate, early, and rapid clinical diagnosis of AMR. This advancement promises to significantly improve patient care and promote antimicrobial stewardship in a timely manner. The main areas of future research directions are to extend the dual ELONA/SERS–RPA platform toward the AMR detection by testing OXA-48 and NDM genes to get the full coverage of the big five carbapenemase genes. Additionally, to employ our sensing platform for the multiplex quantification of different gene ratios in a larger-scale study of clinical specimens to further assess its accuracy in AMR diagnosis in a single-tube test. This would require employing multivariate analysis and machine learning modules to further enhance the detection sensitivity and selectivity for the different genes. Furthermore, the future transition of the RPA assay from a solid phase on magnetic beads into a microfluidic cartridge reaches higher-technology readiness levels. These advancements would enable ultrasensitive, accurate, and multiplex quantification of AMR with a minimal sample preparation time.

## Supporting Information Available

Additional experimental details, results, and discussions including tables for oligonucleotides sequence of the HRP and SERS probes for each gene, gene concentrations for samples used for SERS multiplex detection, and figures for extinction spectra of bare AuNPs and SERS probes, DLS measurements for bare AuNPs and SERS probes, SEM images for SERS probe and magnetic bead, selectivity testing of HRP/SERS probes, and SERS measurement reproducibility (PDF)
