# Supplementary material for: Ultrasensitive Dual ELONA/SERS–RPA Multiplex Diagnosis of Antimicrobial Resistance
Source: Anal Chem. 2024 Jul 8;96(29):12093–101. doi: 10.1021/acs.analchem.4c02165 (PMC11270532; doi:10.1021/acs.analchem.4c02165)
Supplement: Supplementary file 1 — ac4c02165_si_001.pdf [file ac4c02165_si_001.pdf]

**Electronic Supporting Information**

# Ultra-sensitive dual ELONA/SERS - RPA multiplex diagnosis of antimicrobial resistance

Waleed A. Hassanain[a], Christopher L. Johnson[b], Karen Faulds[a*], Neil Keegan[b*] and Duncan Graham[a*]

[a] Department of Pure and Applied Chemistry, Technology and Innovation Centre, University of Strathclyde, Glasgow, G1 1RD, UK. E-mail: duncan.graham@strath.ac.uk, karen.faulds@strath.ac.uk

[b] Translational and Clinical Research Institute, Newcastle University, Newcastle-Upon-Tyne, NE2 4HH, UK. E-mail: neil.keegan@ncl.ac.uk

**Content**

**1. Experimental section**

**1.1. Chemicals and reagents**

**1.2. Preparation of the selective SERS probes**

**1.3. Dual ELONA/SERS - RPA measurements**

**1.4. Control study**

**1.5. Multiplex SERS - RPA measurements**

**2. Results and discussion**

**2.1. Calculations of conversion of template concentrations from mass to molar values**

**2.2. Scanning electron microscopy investigation of SERS probes hybridisation with magnetic beads**

**2.3. Selectivity of HRP/SERS probes and SERS measurement reproducibility**

**Table S1.** Oligonucleotides sequence of the HRP and SERS probes for each gene.

**Table S2.** Gene concentrations for samples used for SERS multiplex detection.

**Figure S1.** Extinction spectra of bare AuNPs and SERS probes of (**a**) VIM, (**b**) KPC and (**c**) IMP.

**Figure S2.** Average results of DLS measurements (n=3) for bare AuNPs and SERS probes of VIM, KPC and IMP. Error bars indicate standard deviations from three measurements.

**Figure S3.** SEM images for (**a**) synthesised VIM SERS probe, (**b**) RPA-amplified target magnetic beads (5 pg VIM) without hybridisation, (**c**) RPA-amplified target magnetic beads (5 pg VIM) hybridised with VIM SERS probe, and (**d**) RPA-amplified target magnetic beads (NTC) hybridised with VIM SERS probe. (**e**) A photographic image for the formed sandwich-like structure solutions with different VIM concentrations after washing.

**Figure S4.** Average ELONA counts (n=3) obtained from testing (**a**) VIM HRP probe, (**b**) KPC HRP probe and (**c**) IMP HRP probe against 50 ng of VIM, KPC and IMP amplified templates. Averaged SERS signal intensity (n=3) of peaks (**d**) 953 $cm^{-1}$ of VIM SERS probe, (**e**) 1194 $cm^{-1}$ of KPC SERS probe and (**f**) 1606 $cm^{-1}$ of IMP SERS probe when tested against VIM, KPC and IMP functionalised magnetic beads subjected to RPA in the presence of 50 ng of template DNA.

**Figure S5.** Reproducibility test by measuring SERS signal intensity of peaks (**a**) 953 $cm^{-1}$ of VIM SERS probe, (**b**) 1194 $cm^{-1}$ of KPC SERS probe and (**c**) 1606 $cm^{-1}$ of IMP SERS probe against 5 pg and 50 ng of each of VIM, KPC and IMP, respectively. Each concentration was tested ten times using different SERS probe batches on different days.

## 1. Experimental section

### 1.1. Chemicals and reagents

TwistAmp® Basic kit was purchased from TwistDx (UK), mono mag streptavidin beads (1 µm) were sourced from Ocean NanoTech (USA). HRP specific probes for each tail (custom designed) and single stranded oligonucleotides (ssDNA) (**Table S1**) for SERS probes synthesis were purchased from Biomers (Germany). DNA LoBind tubes were purchased from Eppendorf (UK). Sodium tetrachloroaurate(III) dihydrate, sodium citrate tribasic dihydrate, sodium chloride, tween-20, phosphate buffer saline (PBS), hybridisation buffer (10x SSC, Triton X-100, sodium dodecyl sulphate and formamide), 1,2-Bis(4-pyridyl)ethylene (BPE) and 4,4'-Dipyridyl (DIPY) were sourced from Sigma Aldrich (UK). 4-(1H-Pyrazol-4-YL)pyridine (PPY) was purchased from Fisher Scientific (UK). TMB peroxidase substrate was sourced from SeraCare Life Sciences (USA).

### 1.2. Preparation of the selective SERS probes

To prepare the selective SERS probes for each target, gold nanoparticles (AuNPs) of ~63 nm size were first prepared using a citrate reduction method. Briefly, all glassware was cleaned first with aqua regia (HCl, HNO3 3:1, v/v), then washed thoroughly with double distilled deionised water (ddH2O). 67.6 mg of sodium tetrachloroaurate(III) dihydrate was added to 500 mL of ddH2O in a 3 necked round bottom flask and heated until boiling.[1] Then, a solution of 45.8 mg of sodium citrate tribasic dihydrate in 7.5 mL ddH2O was added to the flask. The mixture was kept boiling for 15 min with continuous stirring, then allowed to cool to room temperature. The synthesised AuNPs were then characterised using extinction spectroscopy (Cary 60 UV-Vis, Agilent Technologies) and dynamic light scattering (DLS) (Zetasizer, Malvern) measurements (**Figures S1** and **S2**, respectively).

VIM SERS probe was prepared by shaking 900 µL of AuNPs with 100 µL of 1.5 µM Raman reporter PPY for 30 min in a LoBind tube using a compact shaker at speed 200 rpm. Next, the mixture was centrifuged at 4000 rpm for 15 min to remove the excess unattached reporter. After removing the supernatant layer, the pellet was briefly vortexed and re-dispersed into 900 µL ddH2O. 20 µL of 50 µM of VIM ssDNA (Table S1, ESI) was added to the solution and left for 1 hour to bind to the AuNPs surface via Au-S bonding. To improve the DNA loading onto the AuNPs surface, 35 µL of 250 mM sodium citrate buffer solution (pH 3) was added and the solution was shaken for 10 min. This was followed by another addition of 30 µL of 250 mM sodium citrate buffer solution (pH 3) for another 40 min. Next, sodium chloride was added to the mixture to a final concentration of 0.1 M for 45 min. The mixture was then centrifuged at 6000 rpm for 20 min. After removing the supernatant layer, the pellet was briefly vortexed, re-dispersed into 500 µL ddH2O and kept at 4 °C ready for use. KPC and IMP SERS probe solutions were prepared following the same preparation protocol of the VIM SERS probe using

BPE and DIPY as Raman reporters, 50 and 110 µL of 50 µM of KPC ssDNA and IMP ssDNA, respectively. The three prepared SERS probes were then characterised using extinction spectroscopy and DLS measurements (**Figures S1** and **S2**, respectively).

**1.3. Dual ELONA/SERS - RPA measurements**

200 µl of specific HRP reporter probe for each target (**Table S1**) was added to the amplified target-carrying beads (5 fg-50 ng) in each well at a final concentration of 25 nM in 4x SSC. Samples were then incubated with reporter probe for 30 min at room temperature while the plate was shaken at 300 rpm to allow DNA-DNA hybridisation to occur. The samples were then washed three times using 150 µL of PBST per well for 5 min while the plate was shaken at room temperature. After the final washing step, the beads were mixed with 300 µL of TMB peroxidase substrate per well. Blue colouration developed over 5 min before 100 µL of the developed blue solutions were removed from the reaction and placed in a sterile microplate, without the beads, as not to interfere with the colorimetric reading, and the absorbance of the solutions was recorded at 630 nm using a benchtop microplate reader (Tecan infinite M200).

For the SERS measurements, 100 µL of the amplified target-carrying beads (5 fg-50 ng) was incubated with 125 µL of the corresponding SERS probe (**Table S1**) and 30 µL of the hybridisation buffer in LoBind tubes for 30 min at room temperature while shaking at 200 rpm. Mixtures were then washed three times with 300 µL of 0.1M PBS using a magnetic stand for 1 min while shaking at room temperature. Finally, the washing supernatant was removed, the beads were resuspended into 300 µL of 0.1M PBS and transferred to glass vials for the SERS scans using a handheld Raman spectrometer (CBEx, Snowy Range Instruments, USA). All the SERS measurements were carried out using orbital raster scanning (ORS) mode over the range 800-1800 $cm^{-1}$ using Peak 1.3.68 software. A 785 nm laser excitation source with 45 mW of laser power was used with an acquisition time of 1 s. The collected spectra were then baseline corrected using MatLab software. Finally, the average intensity of the SERS signal of each SERS probe was plotted against the corresponding gene concentration (5 fg-50 ng) using a logistic sigmoidal fitting function (Origin 2022).

**1.4. Control study**

To confirm the specificity of the HRP and SERS probes towards their targets, both probes of each target were tested against different genes. VIM HRP probe was tested for hybridisation with 50 ng KPC and IMP amplified templates. KPC HRP probe was tested for hybridisation with 50 ng VIM and IMP amplified templates. IMP HRP probe was tested for hybridisation with 50 ng VIM and KPC amplified templates. Similarly, each SERS probe was tested against different genes-carrying beads following the

same pattern described for the HRP probes testing. The same procedure described in the previous section was repeated and the solutions were scanned using a microplate reader and a handheld Raman spectrometer to monitor the absorbance and SERS signal, respectively.

**1.5. Multiplex SERS - RPA measurements**

In order to perform a multiplex SERS detection of the three genes, 8 different samples of the amplified target-carrying beads (samples 1-8, **Table S2**) were prepared. 100 µL of each sample was incubated with 125 µL of a multiplex SERS probe (mixture of VIM, KPC and IMP SERS probes) and 30 µL of the hybridisation buffer in LoBind tubes for 30 min at room temperature while shaking at 200 rpm. Mixtures containing beads were then washed three times with 300 µL of 0.1M PBS using a magnetic stand for 1 min while shaking at room temperature. Finally, the washing supernatant was removed, the beads were resuspended into 300 µL of 0.1M PBS and transferred to glass vials for the SERS scans using a handheld Raman spectrometer.

## 2. Results and discussion

### 2.1. Calculations of conversion of template concentrations from mass to molar values

The template sequences used in this assay were plasmids containing either the VIM, KPC or IMP gene sequences. The plasmids length was 3553, 3634 and 3493 base pairs (bp), respectively. The molecular mass (Mr) of the plasmids was calculated using the following formula: Mr (kDa) = plasmid length (bp) x base pair weight (660 Da). The molar template concentration can then be calculated in a final reaction volume of 50 µl. For example, Mr of VIM plasmid = 3553 x 660 = 2344.98 kDa. The molar concentration of 50 ng template in 50 µL (1 ng/uL = 0.001 g/L) will be equal to 0.001 ÷ 2344980 = 426 pM. All the other VIM serial concentrations were dropped by 10 times in each step, till reaching the last template mass concentration (5 fg), which will be equivalent to $4.26 \times 10^{-5}$ pM. Similarly, KPC and IMP molar concentrations were calculated using their plasmids length values.

The limit of detection (LOD) values for the three genes using ELONA were < $4.26 \times 10^{-5}$, 0.11 and 0.46 pM for VIM, KPC and IMP, respectively. For the SERS detection, the LOD values were < $4.26 \times 10^{-5}$, 0.09 and 0.06 pM for VIM, KPC and IMP, respectively. The LOD was calculated following the formula: average NTC readings (n=3) + (3 x standard deviation of NTC readings).[2, 3] The lowest LOD observed for VIM indicates low absorbance and SERS signal values for the NTC sample during the assay. This is likely due to minimal primer dimer formation during amplification. In contrast, the NTC values were higher in both the KPC and IMP assays, suggesting that these primer sets are more prone to primer dimerisation. This increased primer dimerisation results in higher NTC values, as long as the reverse primer tail remains free to bind its reporter probe after dimerisation.

### 2.2. Scanning electron microscopy investigation of SERS probes hybridisation with magnetic beads

In order to monitor for the hybridisation events between SERS probes and RPA-amplified target beads, scanning electron microscopy (SEM) investigations were conducted before and after their interaction. The VIM SERS probe was chosen as a representative to observe this hybridization. **Figures S3a** and **S3b** represent the SEM images of the synthesised VIM SERS probe and RPA-amplified VIM functionalised magnetic beads in the presence of 5 pg of VIM template, respectively. Following hybridisation between the VIM SERS probe and the RPA-amplified 5 ng VIM beads, the SEM image revealed the accumulation of the VIM SERS probe on the beads surface, which is attributed to selective hybridisation. Even after washing with 0.1M PBS buffer, the VIM SERS probe remained attached to the beads surface (**Figure S3c**). Minimal binding could be seen between the VIM SERS probe and the RPA-amplified NTC beads, where no target template was added, as illustrated in **Figure S3d**. This phenomenon was evident in the coloration of the sandwich-like structure formed after washing. With

higher target concentrations on the surface of the beads, there were more overhung strands at the 5' end, leading to increased accumulation of the SERS probe on the beads surface and consequently darker solutions (**Figure S3e**).

**2.3. Selectivity of HRP/SERS probes and SERS measurement reproducibility**

To evaluate the selectivity of our methodology, we conducted negative control tests for both the HRP and SERS probes against high concentrations of the cognate and non-cognate amplified target templates, as demonstrators for highly concentrated interfering molecules. This was carried out following the same procedure described for the targets quantification. The VIM HRP probe was subjected to mixing with VIM, KPC and IMP RPA amplicons produced in the presence of 50 ng of template and the resulting solutions were scanned colorimetrically. Even when using the highest concentration of KPC and IMP templates (50 ng), minimal interaction was observed with the VIM HRP probe. The development of a blue coloured solution only occurred when the VIM HRP probe was paired with its cognate target amplicon. This was evidenced by the low counts obtained when testing the VIM HRP probe against non-cognate amplicons, compared to when the same concentration of the cognate target, VIM, was used (**Figure S4a**). Similar findings were observed when testing the KPC and IMP HRP probe against the three genes individually (**Figure S4b,c**). This was attributed to the high selectivity of the HRP probes towards their cognate targets. Using the same approach, we tested the different SERS probes against 50 ng of VIM, KPC and IMP amplified templates and the outcomes were detected by SERS. The SERS analysis yielded a consistent pattern of results similar to those obtained with the HRP probes testing. The SERS signal intensities of VIM, KPC and IMP SERS probes at the peaks 953, 1194 and 1606 $cm^{-1}$, respectively, were insignificantly low, when used with the non-cognate amplicon, compared to those obtained when tested against the matching amplicon (**Figure S4d-f**). These findings demonstrated that the developed HRP and SERS probes were responding only to the corresponding genes, highlighting the high selectivity of the developed methodology.

To ensure the consistency of the SERS measurements, we conducted a series of ten separate SERS measurements on different days, utilizing distinct batches of the synthesized SERS probes. These measurements encompassed both low (5 pg) and high (50 ng) concentrations of each gene. As depicted in **Figure S5a-c**, the minor deviations in the SERS signal intensity of the main characteristic peaks of each SERS probe, between different batches, for the both tested target concentrations indicated the good reproducibility of the developed SERS-based platform. Additionally, the calculated relative standard deviation (RSD) values among the various scans for both high and low concentrations of the three targets ranged from 4.85% to 9.77% (**Figure S5a-c**). Accordingly, the developed SERS-based platform for AMR detection demonstrated good reproducibility across the measurements.

**Table S1**. Oligonucleotides sequence of the HRP and SERS probes for each gene.

| Gene oligonucleotide | Probe | Sequence |
|---|---|---|
| VIM ssDNA | HRP | 5’-HRP-Aminolink-TTG ATA GCA CAG GTC-3’ |
| KPC ssDNA | HRP | 5’-HRP-Aminolink-AAG AGT GTG TCC TGA -3’ |
| IMP ssDNA | HRP | 5’-HRP-Aminolink-AAG GTG TTC CAA CTA -3’ |
| VIM ssDNA | SERS | 5’-TTG ATA GCA CAG GTC-HEG-SH-3’ |
| KPC ssDNA | SERS | 5’-AAG AGT GTG TCC TGA-HEG-SH-3’ |
| IMP ssDNA | SERS | 5’-AAG GTG TTC CAA CTA-HEG-SH-3’ |

**Table S2**. Gene concentrations for samples used for SERS multiplex detection.

| Sample No. | VIM (ng) | KPC (ng) | IMP (ng) |
|---|---|---|---|
| 1 | 50 | 50 | 50 |
| 2 | 50 | 50 | --- |
| 3 | 50 | --- | 50 |
| 4 | --- | 50 | 50 |
| 5 | 50 | --- | --- |
| 6 | --- | 50 | --- |
| 7 | --- | --- | 50 |
| 8 | NTC | NTC | NTC |

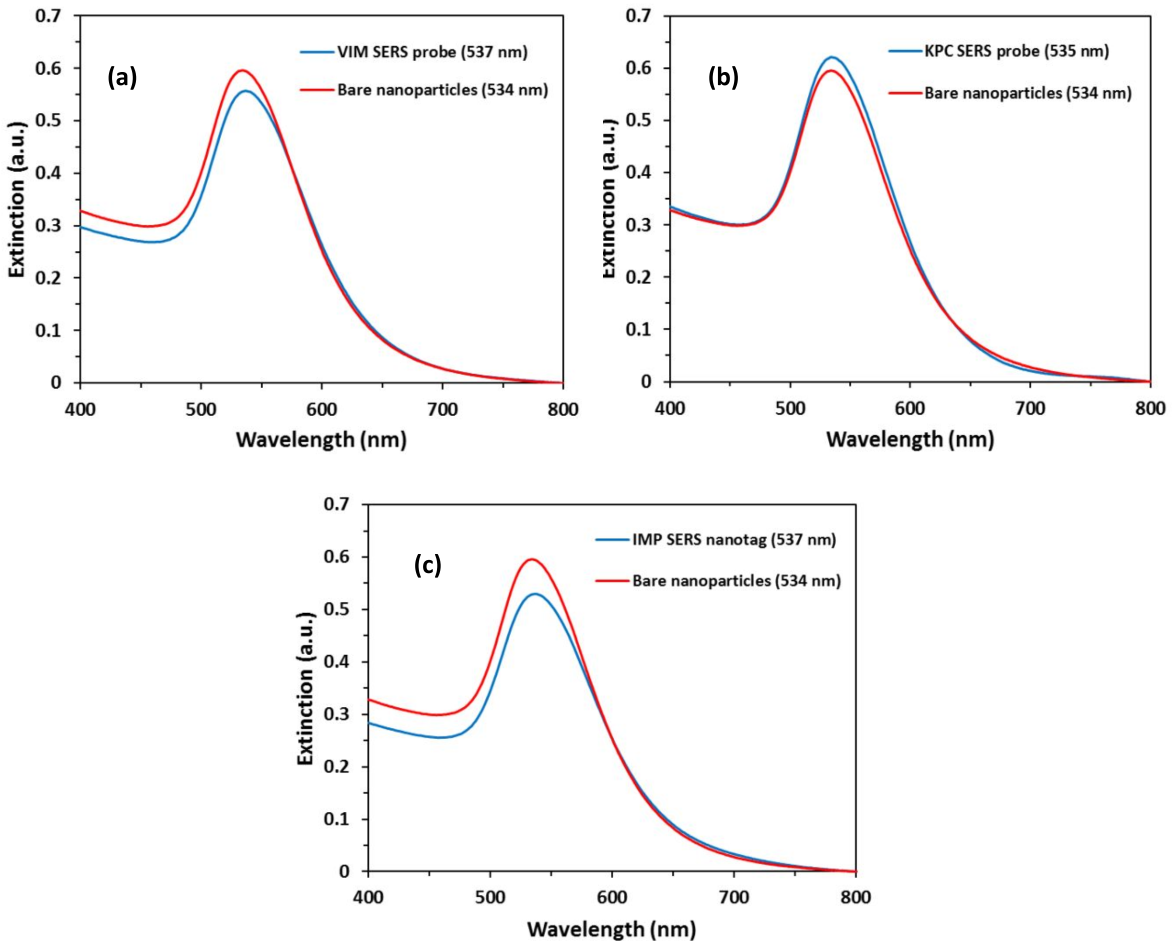


**Figure S1**. Extinction spectra of bare AuNPs and SERS probes of (**a**) VIM, (**b**) KPC and (**c**) IMP.

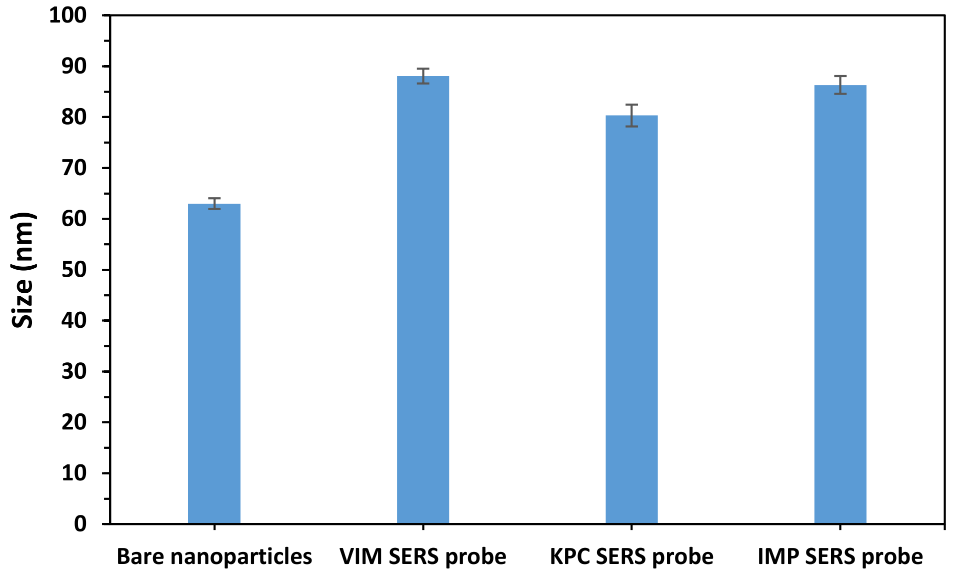


**Figure S2**. Average results of DLS measurements (n=3) for bare AuNPs and SERS probes of VIM, KPC and IMP. Error bars indicate standard deviations from three measurements.

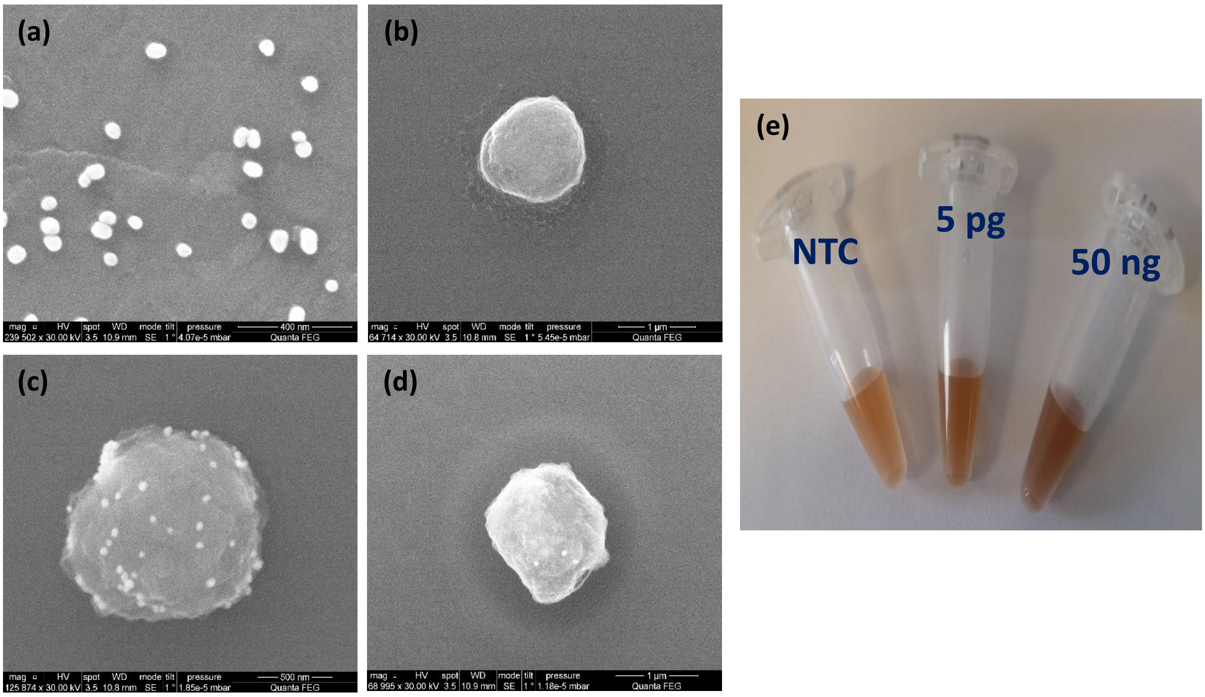


**Figure S3.** SEM images for (**a**) synthesised VIM SERS probe, (**b**) RPA-amplified target magnetic beads (5 pg VIM) without hybridisation, (**c**) RPA-amplified target magnetic beads (5 pg VIM) hybridised with VIM SERS probe, and (**d**) RPA-amplified target magnetic beads (NTC) hybridised with VIM SERS probe. (**e**) A photographic image for the formed sandwich-like structure solutions with different VIM concentrations after washing.

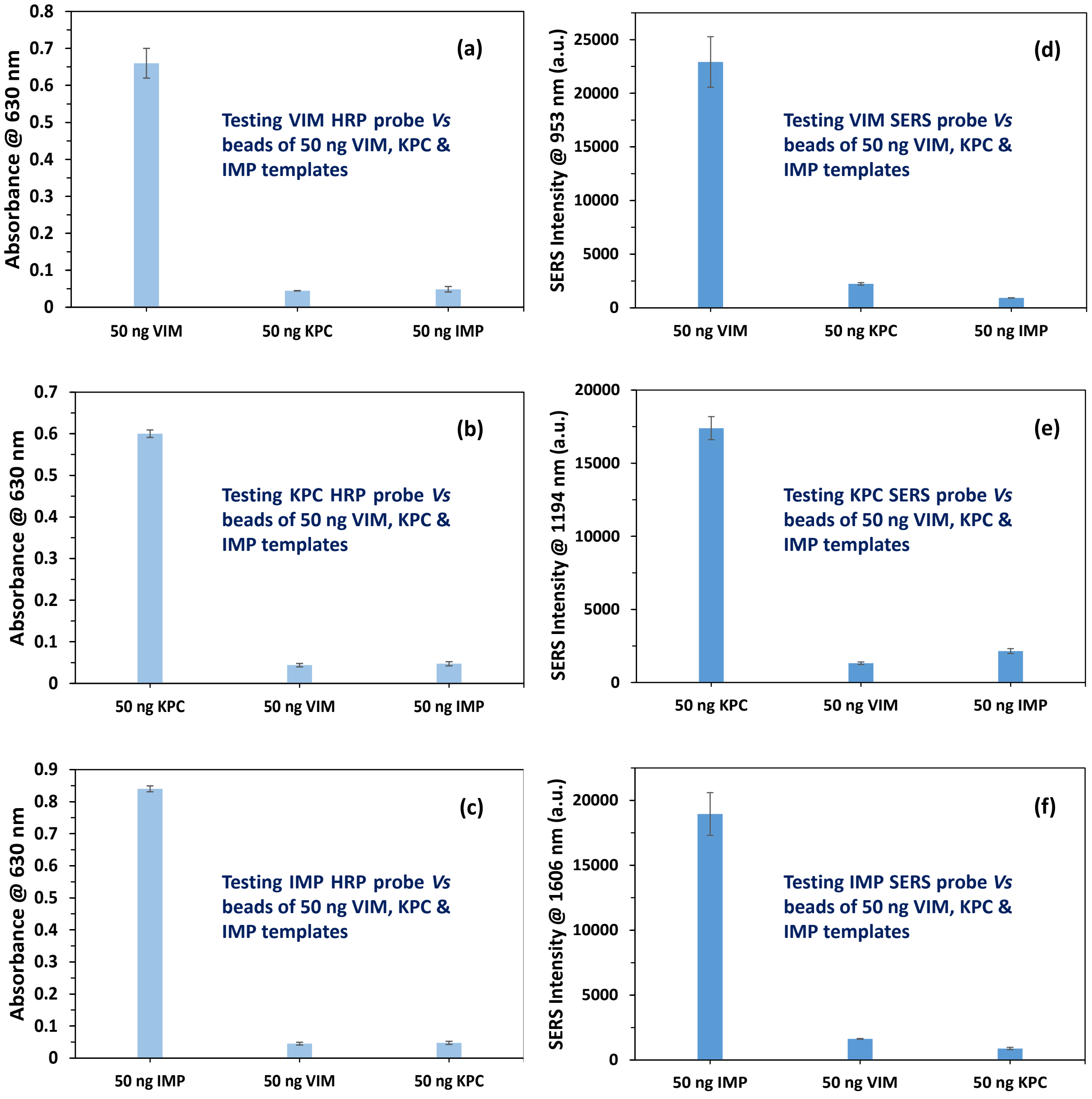


**Figure S4.** Average ELONA counts (n=3) obtained from testing (**a**) VIM HRP probe, (**b**) KPC HRP probe and (**c**) IMP HRP probe against 50 ng of VIM, KPC and IMP amplified templates. Averaged SERS signal intensity (n=3) of peaks (**d**) 953 $cm^{-1}$ of VIM SERS probe, (**e**) 1194 $cm^{-1}$ of KPC SERS probe and (**f**) 1606 $cm^{-1}$ of IMP SERS probe when tested against VIM, KPC and IMP functionalised magnetic beads subjected to RPA in the presence of 50 ng of template DNA. Error bars indicate standard deviations from three measurements. All the colorimetric measurements were carried out using a benchtop microplate reader and the absorbance was measured at 630 nm. All the SERS measurements were carried out using a handheld Raman spectrometer equipped with a 785 nm laser excitation source at 45 mW laser power with an acquisition time of 1s at ORS mode.

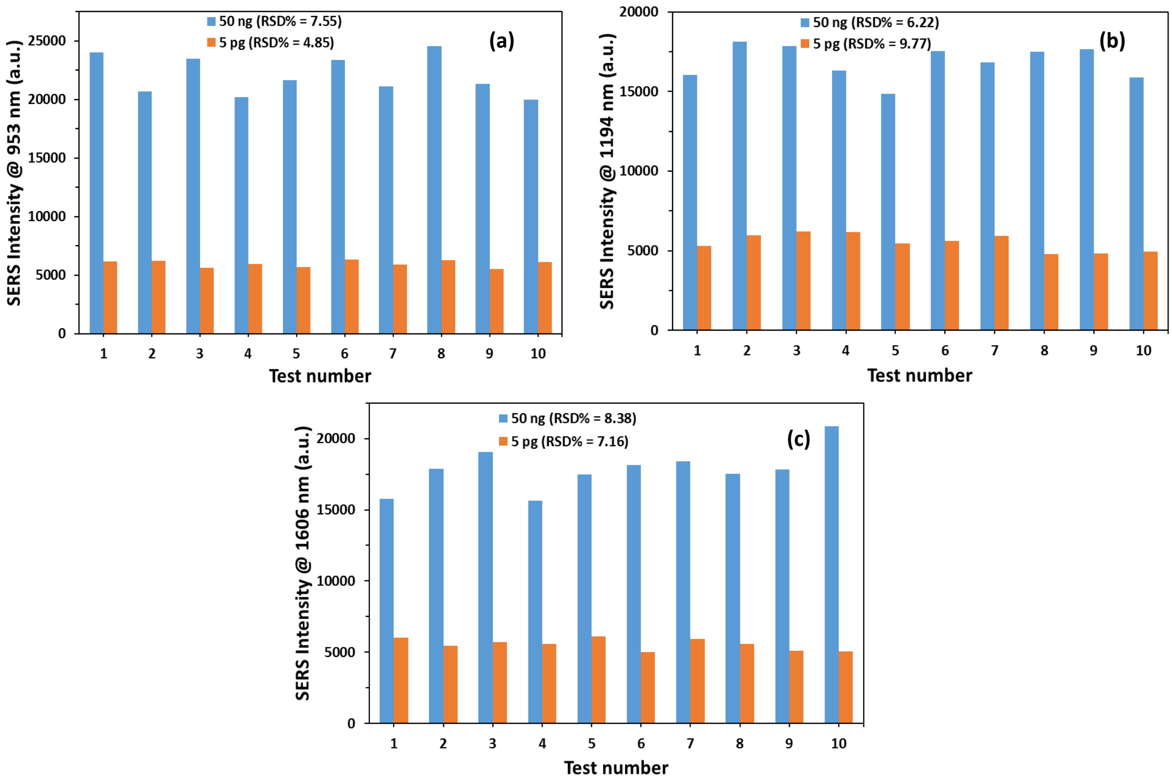


**Figure S5**. Reproducibility test by measuring SERS signal intensity of peaks (**a**) 953 $cm^{-1}$ of VIM SERS probe, (**b**) 1194 $cm^{-1}$ of KPC SERS probe and (**c**) 1606 $cm^{-1}$ of IMP SERS probe against 5 pg and 50 ng of each of VIM, KPC and IMP, respectively. Each concentration was tested ten times using different SERS probe batches on different days. All the SERS measurements were carried out using a handheld Raman spectrometer equipped with a 785 nm laser excitation source at 45 mW laser power with an acquisition time of 1s at ORS mode.

## References

1. Kimling, J.; Maier, M.; Okenve, B.; Kotaidis, V.; Ballot, H.; Plech, A., Turkevich Method for Gold Nanoparticle Synthesis Revisited. *J. Phys. Chem. B* **2006,** *110* (32), 15700-15707.

2. Jauset-Rubio, M.; Svobodová, M.; Mairal, T.; McNeil, C.; Keegan, N.; Saeed, A.; Abbas, M. N.; El-Shahawi, M. S.; Bashammakh, A. S.; Alyoubi, A. O.; O´Sullivan, C. K., Ultrasensitive, rapid and inexpensive detection of DNA using paper based lateral flow assay. *Sci. Rep.* **2016,** *6* (1), 37732.

3. Johnson, C. L.; Setterfield, M. A.; Hassanain, W. A.; Wipat, A.; Pocock, M.; Faulds, K.; Graham, D.; Keegan, N., Multiplex detection of the big five carbapenemase genes using solid-phase recombinase polymerase amplification. *Analyst* **2024,** *149* (5), 1527-1536.
